# Antimicrobial Peptide SAAP‐148‐Functionalized Hydrogels from Photocrosslinkable Polymers with Broad Antibacterial Activity

**DOI:** 10.1002/marc.202400785

**Published:** 2024-11-12

**Authors:** Muhammad Atif, Gizem Babuççu, Martijn Riool, Sebastian Zaat, Ulrich Jonas

**Affiliations:** ^1^ Macromolecular Chemistry Department of Chemistry and Biology University of Siegen Adolf‐Reichwein‐Strasse 2 57076 Siegen Germany; ^2^ Department of Medical Microbiology and Infection Prevention Amsterdam UMC Amsterdam institute for Immunology and Infectious Diseases University of Amsterdam Meibergdreef 9 Amsterdam 1105 AZ The Netherlands; ^3^ Laboratory of Experimental Trauma Surgery Department of Trauma Surgery University Hospital Regensburg Am Biopark 9 93053 Regensburg Germany

**Keywords:** antimicrobial peptides, photocrosslinkable hydrogel, polymer modification, SAAP‐148, wound infection

## Abstract

Antimicrobial peptides (AMPs) are promising alternatives to traditional antibiotics for treating skin wound infections. Nonetheless, their short half‐life in biological environments restricts clinical applicability. Covalent immobilization of AMPs onto suitable substrates offers a comprehensive solution, creating contact‐killing surfaces with long‐term functionality. Here, a copolymer of poly[(hydroxy ethyl acrylamide)‐*co*‐(4‐benzophenone acrylamide)‐*co*‐(pentafluorophenyl acrylate)‐*co*‐(ECOSURF EH‐3 acrylate)], in short poly(HEAAm‐*co*‐BPAAm‐*co*‐PFPA‐*co*‐EH3A), is synthesized by free radical polymerization. Subsequent modification of active ester groups with the amine groups of SAAP‐148, results in a copolymer, that is non‐cytotoxic to human lung fibroblasts. UV photocrosslinking of the benzophenone units yields a polymer network that forms a hydrogel after swelling with aqueous medium. Both the SAAP‐148‐modified polymer in solution and the photocrosslinked hydrogels show good antimicrobial activity against strains of *Escherichia coli*, *Staphylococcus aureus*, *Pseudomonas aeruginosa*, and *Acinetobacter baumannii*, including multidrug‐resistant strains, frequently found in wound infections. The covalent attachment of SAAP‐148 prevents leaching, ensuring sustained antimicrobial activity for at least 48 h in diluted human blood plasma and 14 days in PBS. This prolonged retention of antimicrobial activity in human blood plasma significantly enhances its clinical potential. Overall, this study shows the potential of the AMP‐functionalized photocrosslinkable polymer as antimicrobial wound dressings, providing an effective alternative to antibiotics.

## Graphical abstract

A polymer with covalently attached AMP, SAAP‐148 is developed as potential wound dressing material, which shows antimicrobial activity against various bacteria, including multiresistance strains, for at least 48 hs in diluted human blood plasma.

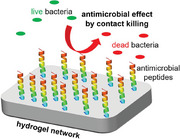

## 1 Introduction

Skin, the largest human organ, makes up 15% of the body weight and serves critical functions in temperature regulation, protection, sensation, maintaining fluid balance, and immune response.^[^ ^1^, ^2^ ^]^ Damage to or malfunction of the skin tissue is typically termed “wound”.^[^ ^3^, ^4^, ^5^ ^]^ Such wounds provide an entry point for bacteria causing infection, which hampers the healing process and can result in patient morbidity and high healthcare costs.^[^ ^6^ ^]^ According to the Wound Healing Society, projections of global expenses for medical care for all wounds ranges from $28.1 to $96.8 billion annually.^[^ ^7^, ^8^ ^]^ If the infected wound is not properly treated, this can result in septicaemia and even death.^[^ ^9^ ^]^ Current wound dressings such as films, sponges, and hydrogels are commonly used for wound care management. These dressings maintain a moist environment to boost the healing process.^[^ ^10^, ^11^ ^]^ Antibiotics are most commonly prescribed as initial treatment for infected wounds, despite them being often ineffective, due to antibiotic resistance, biofilm formation, and inappropriate antibiotic selection.^[^ ^12^, ^13^ ^]^ Effective antibiotic treatment requires the identification of specific microbes for the careful selection of antibiotics. This is typically a very challenging and time‐consuming process, often requiring removal of the wound bandage, interrupting the healing process and imposing patient discomfort.^[^ ^14^ ^]^ Moreover, bacteria present in the infected wound often form biofilms, which are complex structures resistant to both host immune response and antibiotics.^[^ ^15^, ^16^ ^]^ Additionally, systemic antibiotic treatment often fails to deliver adequate concentrations to the infected site, proving to be ineffective against chronic granulating wounds.^[^ ^9^ ^]^ Furthermore, overuse of antibiotics is potentially harmful for gut microbiota.^[^ ^17^ ^]^ Ciprofloxacin for example, which acts against Gram‐negative facultative anaerobes, and levofloxacin, decrease the number of Gram‐positive anaerobes, including bifidobacteria.^[^ ^18^ ^]^ Thus, they may become problematic in preemptive treatment of wound infections. Moreover, wounds colonized with multidrug‐resistant bacteria increase patient morbidity and ultimately compromise wound treatment.^[^ ^19^, ^20^, ^21^ ^]^ It is predicted that without significant interventions, infections caused by antibiotic‐resistant microorganisms could cause 10 million deaths annually worldwide by 2050, surpassing cancer as a leading cause of mortality and adding a global healthcare burden of $100 trillion by that time.^[^ ^22^, ^23^ ^]^

Many efforts have been made to reduce wound infections with methods not relying on antibiotics. In recent years, for example, wound dressings with a slow release of silver (Ag) particles have been developed and used clinically.^[^ ^6^ ^]^ Although Ag particles show good results in eradicating bacteria, Ag ions can be very toxic to healthy tissue cells.^[^ ^24^ ^]^ Moreover, current research also indicates that many bacterial species can develop resistance against Ag particles, raising Ag to the same level as antibiotics regarding antimicrobial resistance.^[^ ^25^ ^]^ Like Ag, most antiseptic agents are nonselective and can become toxic with prolonged use, significantly hindering the wound‐healing process.^[^ ^26^ ^]^

A promising alternative to the above‐mentioned antimicrobial agents are antimicrobial peptides (AMPs), which are natural constituents of the innate immune system of many multicellular organisms with a broad spectrum of antimicrobial activity against pathogens.^[^ ^27^ ^]^ AMPs interact with the bacterial membrane and rapidly compromise it, leading to breakdown of the bacterial cell. This mechanism is less likely to cause bacterial resistance, rendering AMPs a potential alternative to antibiotics or nanoparticles (such as Ag nanoparticles).^[^ ^28^, ^29^, ^30^ ^]^ Furthermore, AMPs have demonstrated therapeutic activity for the treatment of skin and other epithelial injuries through a plethora of additional mechanisms beyond the antimicrobial effect.^[^ ^31^ ^]^ Main examples of AMPs exploited for health care management include pexiganan, under development for the treatment of foot ulcers, and the commercially available talactoferrin, which demonstrates a positive impact on the wound healing process.^[^ ^32^, ^33^ ^]^ Despite these promising properties of AMPs as a potential alternative to conventional antibiotics and antiseptic agents, a significant barrier to bringing them into the clinic is their metabolic degradation.^[^ ^34^ ^]^ For example, dissolved AMPs demonstrated a half‐life of only a few minutes to a few hours when exposed to blood serum or physiological ions.^[^ ^35^, ^36^, ^37^ ^]^ A promising approach to address these challenges of poor peptide stability is the covalent immobilization of AMPs to the wound healing materials to obtain surfaces with long‐lasting antimicrobial activity.^[^ ^38^ ^]^ Many studies have illustrated that AMPs could retain their stability as well as antimicrobial properties when immobilized covalently onto various materials.^[^ ^39^, ^40^, ^41^ ^]^ Moreover, this immobilization approach provides the advantage of substantially reducing cytotoxicity for covalently bound AMPs at high local concentrations compared to freely dissolved AMPs at similar levels.^[^ ^42^, ^43^ ^]^

Considering all of the above‐mentioned issues, management of open skin wound infections remains a significant challenge to clinicians, making prevention the first line of defence. This strategy should be adopted when a wound is at an early stage with microbes having limited proliferation and not yet penetrated deeper into the tissue to trigger a host reaction.^[^ ^44^ ^]^

**Scheme 1:**
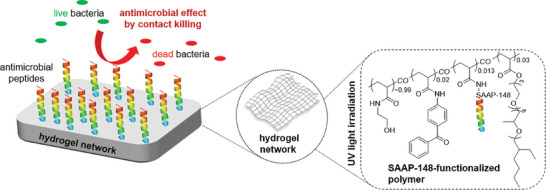
Schematic illustration of the working principle of SAAP‐148‐functionalized hydrogels.

To address this challenge, we have developed an antimicrobial hydrogel with the covalently immobilized synthetic antimicrobial and antibiofilm peptide (SAAP)‐148 (Ac‐Leu‐Lys‐Arg‐Val‐Trp‐Lys‐Arg‐Val‐Phe‐Lys‐Leu‐Leu‐Lys‐Arg‐Tyr‐Trp‐Arg‐Gln‐Leu‐Lys‐Lys‐Pro‐Val‐Arg‐NH2).^[^ ^45^ ^]^ This is the first time SAAP‐148 has been utilized in such a covalently immobilized hydrogel system, offering a novel approach that significantly reduces cytotoxicity while maintaining its potent microbicidal properties. For this, first a copolymer of poly[(hydroxy ethyl acrylamide)‐co‐(4‐benzophenone acrylamide)*‐co‐*(pentafluorophenyl acrylate)*‐co‐*(ECOSURF EH‐3 acrylate)] (in short poly(HEAAm*‐co‐*BPAAm*‐co‐*PFPA*‐co‐*EH3A) was synthesized by free radical polymerization. Poly(hydroxy ethyl acrylamide) is known for a wide range of applications including antifouling coatings, wound dressings, and drug delivery and serves here as main component.^[^ ^46^ ^]^ The synthesized copolymer was subsequently modified with SAAP‐148 in solution by coupling accessible amino groups of SAAP‐148 to the pentafluorophenyl active ester in the polymer backbone. Exposure of the dried polymer film to UV light induced photocrosslinking via the included benzophenone units and the obtained polymer network was swollen in aqueous media to form the antimicrobial hydrogel. The surfactant comonomer ECOSURF EH‐3 acrylate (EH3A) was included to tune the hydrophilic‐hydrophobic balance and to allow interaction with the bacterial cell membrane. A previous study illustrated that the physical embedding of AMPs in a cubic lyotropic liquid crystalline gel as well as their supramolecular interaction on titanium implants supported good bactericidal activity,^[^ ^47^ ^]^ which may be based on leaching of free AMPs from the gel matrix and stimulated our investigation to study the retention of AMP activity after chemical immobilization.^[^ ^48^ ^]^ Thus, the main objective of this study was to evaluate whether the hydrogels with covalently immobilized SAAP‐148 can act as contact‐killing surfaces against bacteria and whether they show favourable properties to be exploited in antimicrobial wound dressings. Moreover, the stability of immobilized SAAP‐148 in the hydrogels in different media, and the cytotoxicity of this SAAP‐148‐modified copolymer, was determined. The concept behind the present research is illustrated in **Scheme** **1**.

## 2 Experimental Section

### 2.1 Materials

All the reagents and chemicals were obtained from commercial sources and used as received unless otherwise noted. N‐(2‐hydroxyethyl)acrylamide (97%), pentafluorophenyl (98%), and laboratory grade ECOSURF EH‐3 (abbreviated here as EH3) were purchased from Sigma–Aldrich. SAAP‐148 was bought from Bisca biochemical (>95%). Azobisisobutyronitrile (AIBN) was recrystallized from methanol (98.5%; VWR Chemicals). Triethyl amine (99.5%; Sigma Aldrich) was distilled before use. 4‐Benzophenone acrylamide (BPAAm) was synthesized according to the literature.^[^ ^49^ ^]^ Roswell Park Memorial Institute (RPMI) medium supplemented with 20 mm Hepes and L‐glutamine, without sodium bicarbonate (further referred to as RPMI) was bought from Sigma–Aldrich. Sodium dodecyl sulfate (SDS) was purchased from Sigma‐Aldrich. Fetal bovine serum (FBS) and Dulbecco's Modified Eagle's Medium (DMEM) were obtained from Thermo Fisher Scientific. Normal human lung fibroblasts (NHLF) were acquired from Lonza, and pooled human plasma (from four donors) was sourced from Sanquin, Amsterdam, The Netherlands. Deionized water (milli‐Q) was used throughout our experiments.

### 2.2 Instruments

#### 2.2.1 Nuclear Magnetic Resonance (NMR)

^1^H NMR (400 MHz) and ^13^C NMR (100 MHz) spectra were recorded on a Bruker AV 400 spectrometer. The spectra were acquired at 25 ± 1 °C using deuterium chloroform (CDCl_3_), deuterium Oxide (D_2_O), and deuterium dimethyl sulfoxide‐d6 (DMSO‐d6) as solvents while tetramethylsilane (TMS) was selected as an internal standard. 1D‐Spectra were analysed with MestreNova 9.

#### 2.2.2 Fourier‐Transform Infrared Spectroscopy (FTIR)

FT‐IR spectroscopy was carried out on a Bruker Tensor 27, having a resolution of 2 cm^−1^ and a total spectral range of 4000–400 cm^−1^.

#### 2.2.3 UV–Visible Spectroscopy

UV–vis spectra were recorded on a BioTek Epoch 2 IVD microplate spectrophotometer (Agilent) in the wavelength range of 300–700 nm and scan rate of 300 nm min^−1^ with baseline correction.

### 2.3 Polymer Synthesis

#### 2.3.1 Synthesis of Monomers

Acrylation of ECOSURF EH‐3 yielded the surfactant monomer EH3A and was performed by the given protocol,^[^ ^50^ ^]^ as described in detail in Section S1 (Supporting Information). The ^1^H NMR spectrum (Figure S1, Supporting Information) shows the successful synthesis of EH3A. ^1^H NMR (500 MHz, CDCl_3_, *T* = 25 °C), δ ppm: 0.84 (t, 6H, 2x‐CH_3_), 1.12–1.25 (m, 20H, 4x‐CH_3_ and 4x‐CH_2_‐), 1.47 (m, 1H, ─CH─), 3.28–3.72 (m, 22H, 4x‐CH‐ and 9x‐CH_2_─O─), 4.30 (m, 2H, ─CH_2_─O─), 5.81 (m, 1H, ─CH═CH_2_), 6.14 (m, 1H, ─CH═CH_2_), 6.41 (m, 1H, ─CH═CH_2_). Moreover, integration of the ^1^H NMR peaks in the range of 5.8–6.5 ppm indicates a product purity of ≈75%. The remaining 25% likely consists of unreacted starting materials (educts) and residual solvents. Consequently, the integral values may deviate slightly from the theoretical calculations. However, this impurity level is not expected to significantly impact the subsequent polymerization process. 4‐Benzophenone acrylamide (BPAAm) as a photocrosslinker monomer was synthesized as reported in the literature,^[^ ^49^ ^]^ and described in detail in Section S2 (Supporting Information). The ^1^H NMR spectrum (Figure S2, Supporting Information) shows the successful synthesis of BPAAm. ^1^H NMR (500 MHz, CDCl_3_, *T* = 25 °C), δ ppm: 5.70 (dd, 1H, H_2_C═CHCONH─), 6.42 (dd, 1H, H_2_C═CH─CONH─), 6.64 (dd, 1H, H_2_C═CH─CONH─), 7.42 (m, 2H, ‐arom), 7.53 (m, 1H, ‐arom), and 7.74 (m, 6H, ‐arom). Pentafluorophenyl acrylate (PFPA) as an active ester monomer was synthesized as reported previously^[^ ^51^ ^]^ and described in detail in Section S3 (Supporting Information). The ^1^H NMR spectrum (Figure S3, Supporting Information) shows the successful synthesis of PFPA. ^1^H NMR (500 MHz, CDCl_3_, *T* = 25 °C), δ ppm: 6.33 (dd, 1H, ─CH═CH_2_), 6.38 (dd, 1H, ─CH═CH_2_), and 6.60 (dd, 1H, ─CH═CH_2_).

#### 2.3.2 Synthesis of Poly(HEAAm‐*co*‐BPAAm‐*co*‐PFPA‐*co*‐EH3A) and Its Modification with SAAP‐148

**Scheme 2:**
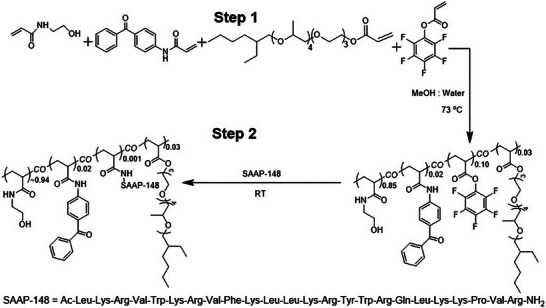
Synthesis of poly(HEAAm‐co‐BPAAm‐co‐SAAP‐148‐co‐EH3A). Room temperature is abbreviated as RT.

A copolymer was synthesized containing hydroxy ethyl acrylamide (HEAAm) as a biocompatible main monomer, BPAAm as photocrosslinker monomer to form a hydrogel, EH3A as surfactant for hydrophilic and hydrophobic balance and amphiphilicity, and PFPA as an active ester monomer to enable post‐polymerization modification (PPM). The resultant copolymer (poly(HEAAm‐*co*‐BPAAm‐*co*‐PFPA‐*co*‐EH3A)) was modified with the antimicrobial peptide SAAP‐148 by using active ester chemistry to form poly(HEAAm‐*co*‐BPAAm‐*co*‐SAAP‐148‐*co*‐EH3A). In the synthesis protocol (Step 1 of **Scheme** **2**), HEAAm (500 mg, 4.41 mmol), EH3A (72.60 mg, 0.13 mmol), BPAAm (22.20 mg, 0.08 mmol), PFPA (73.60 mg, 0.30 mmol), and AIBN (7.25 mg, 0.44 mmol, 1 mol%) were transferred to a Schlenk tube with a magnetic stirring bar. The mixture was filled with 10 mL of methanol, purged with argon for 30 min, and heated at 65 °C for 24 h. After precipitation in diethyl ether and overnight vacuum drying, the white product, named poly(HEAAm‐*co*‐BPAAm‐*co*‐PFPA‐*co*‐EH3A) (poly(HEAAm‐*co*‐PFPA)) was obtained with around 67% yield. ^1^H NMR, ^19^F NMR, and FTIR corroborated the successful synthesis of the target polymer, as provided in the SI (Figures S4–S6, Supporting Information). ^1^H NMR (500 MHz, DMSO, *T* = 25 °C), δ ppm: 0.8–2.25 (EH3A and polymer backbone), 2.8–3.7 (ethyl group of HEAAm), 4.6–5.2 (─OH protons of HEAAm), 7.2–8.0 (aromatic proton of BPAAm side group units and amide protons of HEAAm). ^19^F NMR (500 MHz, DMSO), δ (ppm): −162/−164 (2F) (pentafluorophenyl, meta), −156/−158 (1F) (pentafluorophenyl, para), −150/−154 (2F) (pentafluorophenyl, ortho). FT‐IR (cm^−1^): 1100–1270 (C─F), 1600–1650 (aromatic ring), 1725–1750 (C═O).

In the next step (Step 2 of Scheme 2), 100 mg (0.42 mmol) of poly(HEAAm‐*co*‐BPAAm‐*co*‐PFPA‐*co*‐EH3A) were dissolved in 5 mL of an 80:20 water‐dioxane solvent mixture. To this solution, 0.88 mL of SAAP‐148 (1 mg mL^−1^) and 100 µL of triethylamine were added. The reaction was carried out at 40 °C for 48 h. After this period, 75 µL of ethanolamine (10 equivalents) was added to the reaction mixture and the reaction was continued for an additional 24 h at room temperature. Upon completion, the reaction mixture was freeze dried. Subsequently, the freeze‐dried product was subjected to the dialysis for 24 h. After purification (dialysis), the product was freeze‐dried to yield a final polymer called poly(HEAAm‐*co*‐BPAAm‐*co*‐SAAP‐148‐*co*‐EH3A) poly(HEAAm‐*co*‐SAAP‐148). The obtained products poly(HEAAm‐*co*‐PFPA) and poly(HEAAm‐*co*‐SAAP‐148) were characterized by ^1^H NMR (Figure S7, Supporting Information), and ^19^F NMR (Figure S8, Supporting Information). Due to the low SAAP‐148 content (0.001 mol%), its ¹H NMR signal identification (Figure S7, Supporting Information) is challenging. However, resonances at 2.72 ppm, 2.80–3.82 ppm, and 4.63–5.31 ppm might be indicative of the presence of SAAP‐148 in the polymer backbone. ¹⁹F NMR definitively confirms the modification (Figure S8, Supporting Information). The absence of a signal compared to premodification (Figure S5, Supporting Information) signifies complete consumption of PFPA ester groups, suggesting successful SAAP‐148 conjugation.

### 2.4 Hydrogel Formation

The polymer solution (25 mg mL^−1^ in methanol and water (90:10; v/v)) was prepared in a petri dish and dried over the hotplate at 40 °C overnight. The dried polymer layer was irradiated inside an UV photocrosslinker (ultraviolet crosslinker CL‐1000; UVP) at a wavelength of 365 nm (1 h = 13.0 J cm^−^^2^) for 30 min to form the crosslinked polymer network structure. This photocrosslinked polymer network structure was swollen in water at room temperature for 30 min to form the hydrogel.

### 2.5 WST‐01 Assay

According to ISO 10993–5 standards for cytotoxicity testing, fibroblasts are recommended, specifically the L929 mouse fibroblast cell line. In order to provide a more directly relevant assessment of cytotoxicity in human cells, we opted for the human NHLF fibroblast cell line. In this experiment, 0.1–5 × 10^4^ NHLF cells per well were cultured in a 96‐well mL plate with a final volume of 100 µL of DMEM with 2% FBS. The cells were incubated for 24 h, after which the medium was replaced with 100 µL of fresh DMEM with 2% FBS. Twofold serial dilutions of SAAP‐148‐free and SAAP‐148‐modified polymers with a concentration 25 mg mL^−1^ (2 replicates each; final volume of 100 µL) were added to separate wells and incubated for an additional 24 h. Subsequently, 10 µL of WST‐1 reagent was added to each well and incubated for 30 min. The colour of the media changed from pink to yellowish after 30 min. The reaction was then stopped by adding 10 µL of 1% SDS to each well and shaking the plate for 1 min to mix the contents. Absorbance of the (untreated) control and treated samples was measured using a microplate reader (Synergy H1, Biotek, USA) at a wavelength of 400 nm. One hundred microliter culture medium with 10 µL WST‐1 was used as blank control in separate wells.

### 2.6 Bacterial Culture

Prior to the experiment, clinical and laboratory strains of *Escherichia coli* (*E. coli*) (PC 1568, PC 2348AR4, DH5α, B12C1, DC10B,^[^ ^52^ ^]^ TOP10,^[^ ^53^ ^]^ ATCC 8739,^[^ ^54^ ^]^ and ML35 (ATCC 43 827)),^[^ ^55^ ^]^ *Staphylococcus aureus* (*S. aureus*) strain JAR060131,^[^ ^56^ ^]^ a standard laboratory strain of *Pseudomonas aeruginosa* (*P. aeruginosa*) PAO1, and a multidrug‐resistant strain of *Acinetobacter baumannii* (*A. baumannii*) RUH875^[^ ^57^ ^]^ from frozen stocks were grown at 37 °C on blood agar plates for 16–18 h. From each plate, overnight cultures were prepared in lysogeny broth (Oxoid) for all *E. coli* strains, and in tryptic soy broth (Oxoid) for *S. aureus*, *P. aeruginosa*, and *A. baumannii*, and incubated at 37 °C for 18–24 h at 120 rpm. Prior to each experiment, bacteria were cultured to the mid‐logarithmic growth phase in their respective media. Bacteria were washed twice with phosphate‐buffered saline (PBS; pH 7.0) and diluted in RPMI to 1 × 10^6^ colony forming units (CFU) mL^−1^ based on the optical density at 620 nm.

### 2.7 Analysis of Microbicidal Properties

#### 2.7.1 Qualitative Analysis

For qualitative analysis, 100 µL of the bacterial suspension was spread onto tryptone soy agar plates, covering the entire agar surface to ensure confluent growth. The plates were left for several minutes to dry. Subsequently, 10 µL aliquots of the polymer solutions were deposited on the agar surface at marked locations (number of replicates *n* = 2). The plates were then incubated overnight at 37 °C. The next day, inhibition zones were recorded to qualitatively analyse the antimicrobial properties of the SAAP‐148‐modified polymer solutions.

#### 2.7.2 Quantitative Analysis

Quantitative analysis of antimicrobial properties was performed by using a modified Miles and Misra method.^[^ ^58^ ^]^ In this analysis, 100 µL of polymer solutions were added to each well of a 96‐well plate (number of replicates *n* = 2), followed by the addition of 100 µL of bacterial suspension (final concentration of 5 × 10^5^ CFU mL^−1^). The plates were then incubated with shaking overnight at 37 °C. Then, three 10 µL aliquots of each suspension and tenfold serial dilutions were cultured on blood agar plates overnight at 37 °C. The aliquots were pipetted up and down multiple times at each dilution step to ensure thorough and reproducible mixing. Finally, the number of colonies were counted.

### 2.8 Stability Test of Chemically Immobilized SAAP‐148 in the Hydrogel

In this test, SAAP‐148‐modified hydrogel (Poly(HEAAm‐*co*‐SAAP‐148)), and SAAP‐148‐free hydrogel (PHEAAm) as a control were casted in the wells of a 96‐well plate. For physical embedding of SAAP‐148, the PHEAAm hydrogel was incubated with a 10 µm solution of SAAP‐148 in water. Subsequently, 100 µL of PBS (pH 7) was added to both types of hydrogels, and samples were incubated at 37 °C for up to 14 days (number of replicates *n* = 2). At each time point (1 h, 1 day, 7 days, and 14 days) all liquid was removed from the wells for later bactericidal activity assessment (see below) and then the hydrogels were rinsed three times with PBS. Afterward, 100 µL of *E. coli* DH5α inoculum suspension was added and incubated overnight. Subsequently, three 10 µL aliquots from each well and their serial tenfold dilutions were cultured on blood agar plates overnight at 37 °C. Finally, the number of colonies were counted to determine how long each hydrogel type retained its bactericidal properties.

Moreover, stability of SAAP‐148 attachment in the polymer backbone after 7 days of incubation in PBS was further assessed. For this analysis, 100 µL of PBS aliquots used for rinsing the hydrogel (with chemically immobilized SAAP‐148) were mixed with 100 µL of *E. coli* DH5α inoculum suspension (final concentration of 5 × 10^5^ CFU mL^−1^) and incubated overnight. From this stock suspension, a dilution series was prepared and three 10 µL aliquots were pipetted onto blood agar plates, incubated overnight, and then the number of colonies were counted. PBS was taken as control.

### 2.9 Determination of Antimicrobial Properties of SAAP‐148‐Modified Hydrogel in Human Blood Plasma

For this analysis, SAAP‐148‐modified hydrogel was casted in a 96‐well plate, as discussed above. To each well, 100 µL of 20% human plasma diluted in sterile milli‐Q water was added and samples were preincubated with the plasma at 37 °C from 1 to 48 h. At each time point, the diluted plasma was removed from duplicate samples of the hydrogels, and they were rinsed three times with 100 µL volumes of PBS. Hundred microliter of *E. coli* DH5α inoculum suspension was added, and the hydrogels were incubated overnight at 37 °C. Subsequently, three separate 10 µL aliquots of each suspension were tenfold serially diluted in PBS and plated onto blood agar plates with overnight incubation. Finally, the number of CFU were determined to investigate how long the SAAP‐148‐modified hydrogels retain their bactericidal activity in the presence of human blood plasma. SAAP‐148‐free hydrogel (PHEAAm) was used as a control.

## 3 Results and Discussion

### 3.1 Synthesis of SAAP‐148‐Modified Single‐Chain Polymers and Polymer Network Hydrogels

Acrylate‐based monomers were synthesized according to the literature for the preparation of the copolymer poly(HEAAm‐*co*‐BPAAm‐*co*‐PFPA‐*co*‐EH3A), which can be both photocrosslinked and further functionalized with the amino groups of SAAP‐148 by a PPM reaction. The monomers included PFPA as an activated ester for PPM,^[^ ^51^ ^]^ EH3A as surfactant monomer to introduce an amphiphilic character,^[^ ^50^ ^]^ BPAAm as photocrosslinking monomer,^[^ ^49^ ^]^ and HEAAm as hydrophilic and biocompatible main monomer.^[^ ^59^ ^]^ The copolymer was obtained by free radical polymerization and characterized using ¹H NMR, ¹⁹F NMR, and FTIR spectroscopy (Figures S4, S5, and S6, Supporting Information, respectively). The ¹H NMR spectrum documents the successful incorporation of all monomers by the presence of signals corresponding to the polymer backbone and EH3A in the range of 0.8–2.25 ppm, the ethyl group of HEAAm at 2.8–3.7 ppm, the ─OH protons of HEAAm at 4.6–5.2 ppm, and the aromatic protons of BPAAm side groups along with the amide protons of HEAAm at 7.2–8.0 ppm. Moreover, the three distinct chemical shifts observed in the ^19^F NMR spectra at −162/−164 ppm (meta), −156/−158 ppm (para), and −150/−154 ppm (ortho) are characteristic of a pentafluorophenyl ring present in the copolymer. The FTIR spectrum displays a strong band in the range of 1100–1270 cm^−1^, indicating C─F stretching vibrations which further supports the presence of a fluorinated moiety. The band observed between 1600–1650 cm⁻¹ is consistent with aromatic ring stretching, and the band at 1725–1750 cm⁻¹ suggests the presence of a carbonyl group (C═O).

The copolymer with active ester groups was subsequently functionalized with SAAP‐148 via its amine groups through PPM. The successful modification was corroborated by ¹H NMR and ¹⁹F NMR spectroscopy (Figures S7 and S8, Supporting Information, respectively). Due to the low fraction (0.001 mol%) of SAAP‐148 within the polymer backbone and overlap of the large number of signals of AMP with the signals of the polymer chains, identification of the protein signals in the ^1^H NMR spectrum becomes challenging. However, the ¹H NMR spectrum of the SAAP‐148‐modified copolymer shows additional resonances at 2.72, 2.80–3.82, and 4.63–5.31 ppm compared to the spectrum before modification, which can be attributed to the immobilized SAAP‐148 within the polymer backbone (Figure S7, Supporting Information) and expected functionalization yield is around 80% with respect to the active ester monomer contents. Successful PPM with SAAP‐148 is further indicated by ¹⁹F NMR spectroscopy, as the PPM spectra (Figure S8, Supporting Information) lack any signal compared to the premodified polymer (Figure S5, Supporting Information), signifying the complete consumption of active ester groups on the original copolymer. Yet, since ethanolamine is used in a second step during PPM to quench the remaining active ester groups, not all active sites may have been modified with SAAP‐148.

For the preparation of the hydrogel films, the SAAP‐148‐modified copolymer was dissolved in water, deposited into petri dishes or 96‐well plates, respectively, and then dried to form a neat polymer layer. The dried films were subsequently exposed to UV light to induce crosslinking and create a surface‐attached polymer network,^[^ ^60^ ^]^ which can be swollen by water to form a hydrogel. The mechanism of photocrosslinking and formation of hydrogel is shown in Figure S9 (Supporting Information). The resulting hydrogel immobilized in the 96‐well plate was then employed for microbiological experiments to investigate the stability of covalently immobilized SAAP‐148 and the antimicrobial properties of the SAAP‐148‐modified hydrogel in the presence of human blood plasma.

Based on earlier observations, AMPs physically embedded in a hydrogel matrix possess high antimicrobial activity,^[^ ^48^ ^]^ however they can be leaching out to the surrounding environment in an uncontrolled way. The hypothesis in our current study was that covalent immobilization of SAAP‐148 would prevent leaching out while retaining the antimicrobial activity via a contact killing mechanism.

### 3.2 Cytotoxicity Testing of Poly(HEAAm‐*co*‐SAAP‐148)

**Figure 1:**
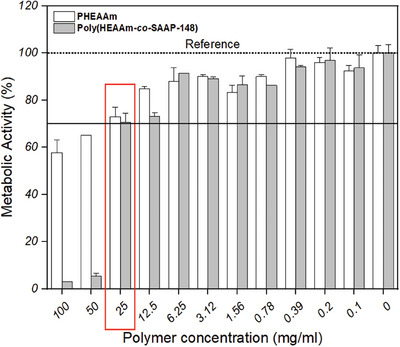
Assessment of metabolic activity by the WST‐1 assay for NHLF cells in contact with solutions of single chain polymers without (PHEAAm) and with covalently attached antimicrobial peptides (poly(HEAAm‐co‐SAAP‐148)). Solid black line indicates 70% cut‐off value for viability. Values are mean percentage metabolic activity + SD, duplicate samples were used (*n* = 2).

The viability of normal human lung fibroblasts (NHLF) exposed to concentration series of poly(HEAAm*‐co‐*SAAP‐148) and SAAP‐148‐free PHEAAm polymer was studied with the WST‐1 metabolic activity assay. This method is based on the conversion of the tetrazolium salt WST‐1 to formazan by mitochondrial dehydrogenases. The active mitochondria of viable cells produce dehydrogenase enzymes for the WST‐1 reaction. The amount of formazan produced can be quantified by measuring the absorbance of the dye at a specific wavelength The results illustrated that more than 70% of the cells in the 96 well plate remained metabolically active when in contact with the SAAP‐148‐modified polymer solution at a concentration up to 25 mg mL^−1^ (**Figure** **1**). This indicates low cytotoxicity according to the international standard test ISO 10993–5:2009 for medical devices.^[^ ^61^ ^]^ This property makes SAAP‐148‐immobilized hydrogels suitable antibacterial surfaces for preventing bacterial infections in wound management, while not harming healthy tissue cells.

Moreover, the metabolic activity after exposure to either poly(HEAAm‐*co*‐SAAP‐148) or SAAP‐148‐free PHEAAm polymers decreased with increasing polymer concentration. For poly(HEAAm‐*co*‐SAAP‐148), the higher concentration of SAAP‐148 exhibited increased cytotoxicity, likely due to its hydrophobic nature.^[^ ^62^, ^63^ ^]^ In the case of SAAP‐148‐free PHEAAm polymers, an increase in concentration concomitantly led to a higher benzophenone content, which correlated with decreased metabolic activity of the cells, consistent with previous findings.^[^ ^64^ ^]^

### 3.3 Qualitative Analysis of Microbicidal Properties of SAAP‐148‐Modified Single‐Chain Polymers in Solution

**Figure 2:**
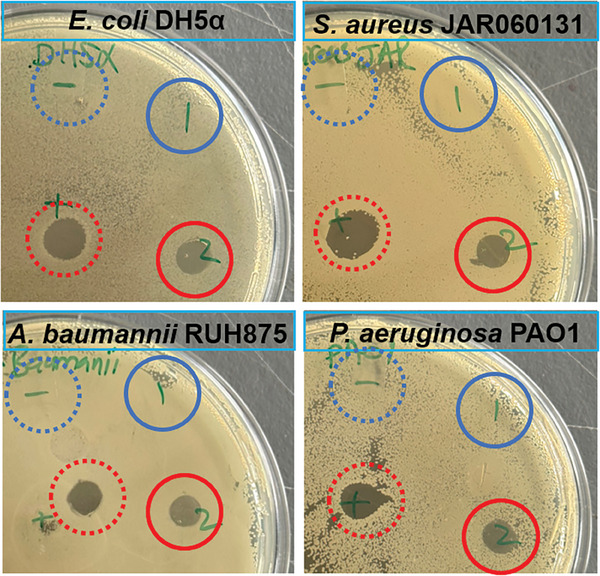
Qualitative analysis of antimicrobial properties of SAAP‐148‐modified polymer in solution against *E. coli*, *S. aureus*, *P. aeruginosa*, and *A. baumannii*. 1) PHEAAm (without AMP) as a reference polymer in blue solid circles. 2) poly(HEAAm‐co‐SAAP‐148) polymer in red solid circles. (−) PBS as negative control in blue dotted circles. (+) 120 µm SAAP‐148 solution as positive control in red dotted circles. Duplicates samples were used (*n* = 2).

An agar diffusion assay was performed to analyse the antimicrobial properties of the SAAP‐148‐modified polymer in solution against *E. coli*, *S. aureus*, *P. aeruginosa*, and *A. baumannii*. In this analysis, PBS was taken as negative control (blue dotted circles in **Figure** **2**) and 120 µm of SAAP‐148 solution was taken as positive control (red dotted circles in Figure 2). No inhibition zone was observed for PBS and for the SAAP‐148‐free polymer (poly(HEAAm‐*co*‐BPAAm‐*co*‐EH3A)) (PHEAAm) in aqueous solution (blue solid circles in Figure 2). In contrast, a solution of SAAP‐148‐modified polymer (poly(HEAAm‐*co*‐SAAP‐148)) in water caused clearly visible inhibition zones (red solid circles in Figure 2) with all the tested bacterial species. This demonstrates the high effectiveness of the SAAP‐148‐modified polymers to inhibit the growth of all tested bacterial strains. The results further demonstrate that coupling of SAAP‐148 to our polymer backbone does not eliminate its antimicrobial activity, enabling it to retain its efficacy against a broad spectrum of tested bacterial species.

### 3.4 Quantitative Analysis of Microbicidal Properties of SAAP‐148‐Modified Single‐Chain Polymer in Solution

**Figure 3:**
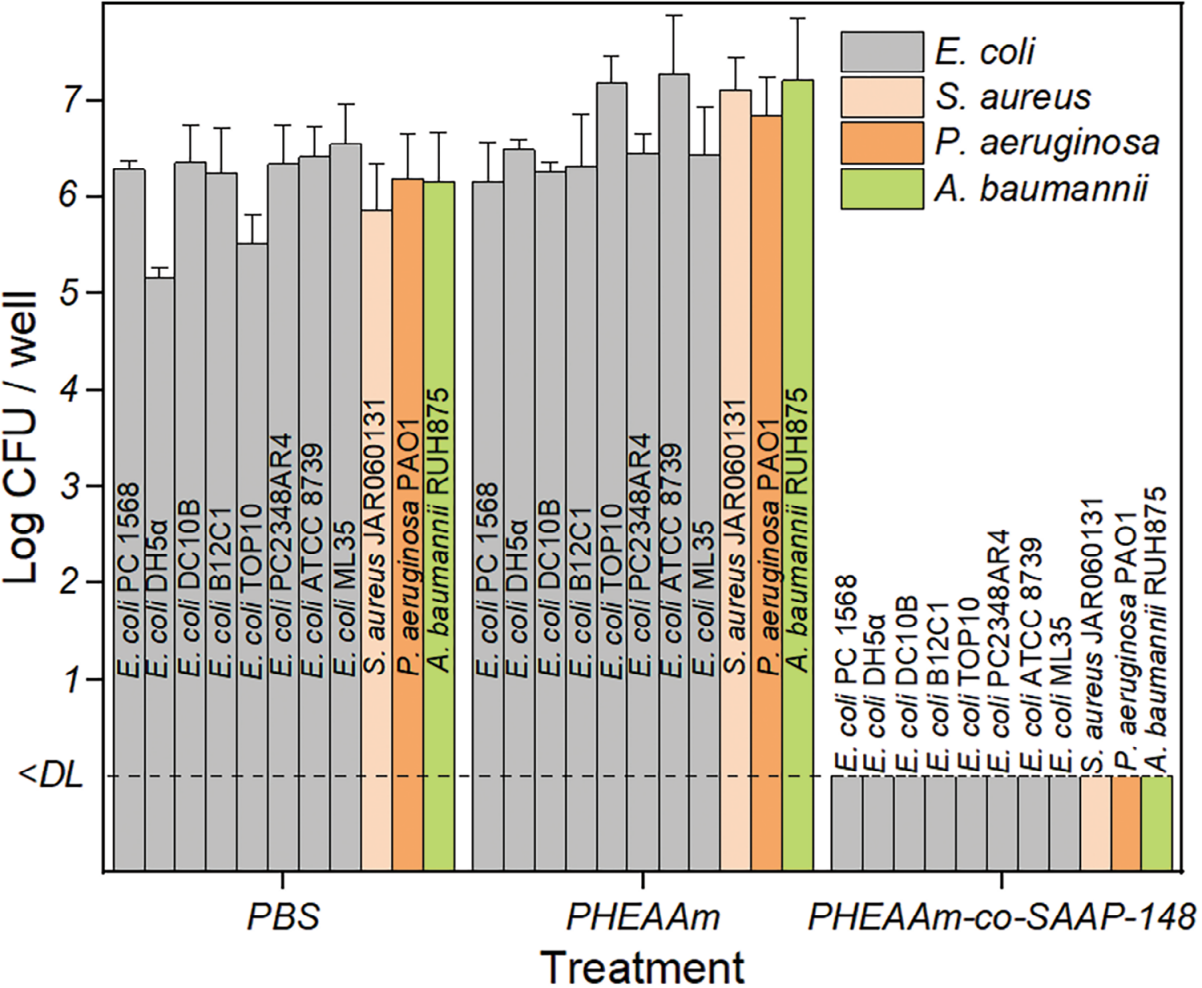
Modified Miles and Misra method of the microbicidal properties of single chain polymer Poly(HEAAm‐co‐SAAP‐148) in solution. The incubation time was 24 h. The inoculum was 10^5^ CFU per well. Values are mean number of CFU +SD, duplicate samples were used (*n* = 2). The lower limit of detection (DL) is 6.6 CFU.

The quantitative analysis of antimicrobial properties of the SAAP‐148‐modified polymer in solution was performed with a modified Miles and Misra method against eight strains of *E. coli*, one strain of *S. aureus*, one strain of *P. aeruginosa*, and one strain of *A. baumannii* (**Figure** **3**). To determine the impact of the SAAP‐148 modification, the antimicrobial activity of SAAP‐148‐modified polymer in solution was compared with both an unmodified (SAAP‐148‐free) polymer solution and a PBS control, as demonstrated in Figure 3. The high number of CFU, between 10^5^ and 10^7^ CFU per well, observed for the reference polymer samples without SAAP‐148 (PHEAAm) and PBS indicated no effect on the bacterial growth of all tested strains. On the other hand, full eradication of the inoculum of all tested strains of *E. coli*, *S. aureus*, *P. aeruginosa*, and *A. baumannii* was observed for SAAP‐148‐modified polymers in solution. In addition, the SAAP‐148‐modified polymer in solution caused eradication of *E. coli* DH5α and *S. aureus* JAR060131 after just 2 h of incubation time, as illustrated in Figure S10 (Supporting Information). These observations correlate well with and extend the results of the agar diffusion assay (Section 3.3).

Overall, the results of this study indicated that the SAAP‐148‐modified polymer in solution exhibits robust antimicrobial properties, effectively eliminating a diverse range of bacterial species and strains in a short time and demonstrated high potential for various biomedical and industrial applications.

### 3.5 Stability Test of Covalently Immobilized SAAP‐148 in the Hydrogel

**Figure 4:**
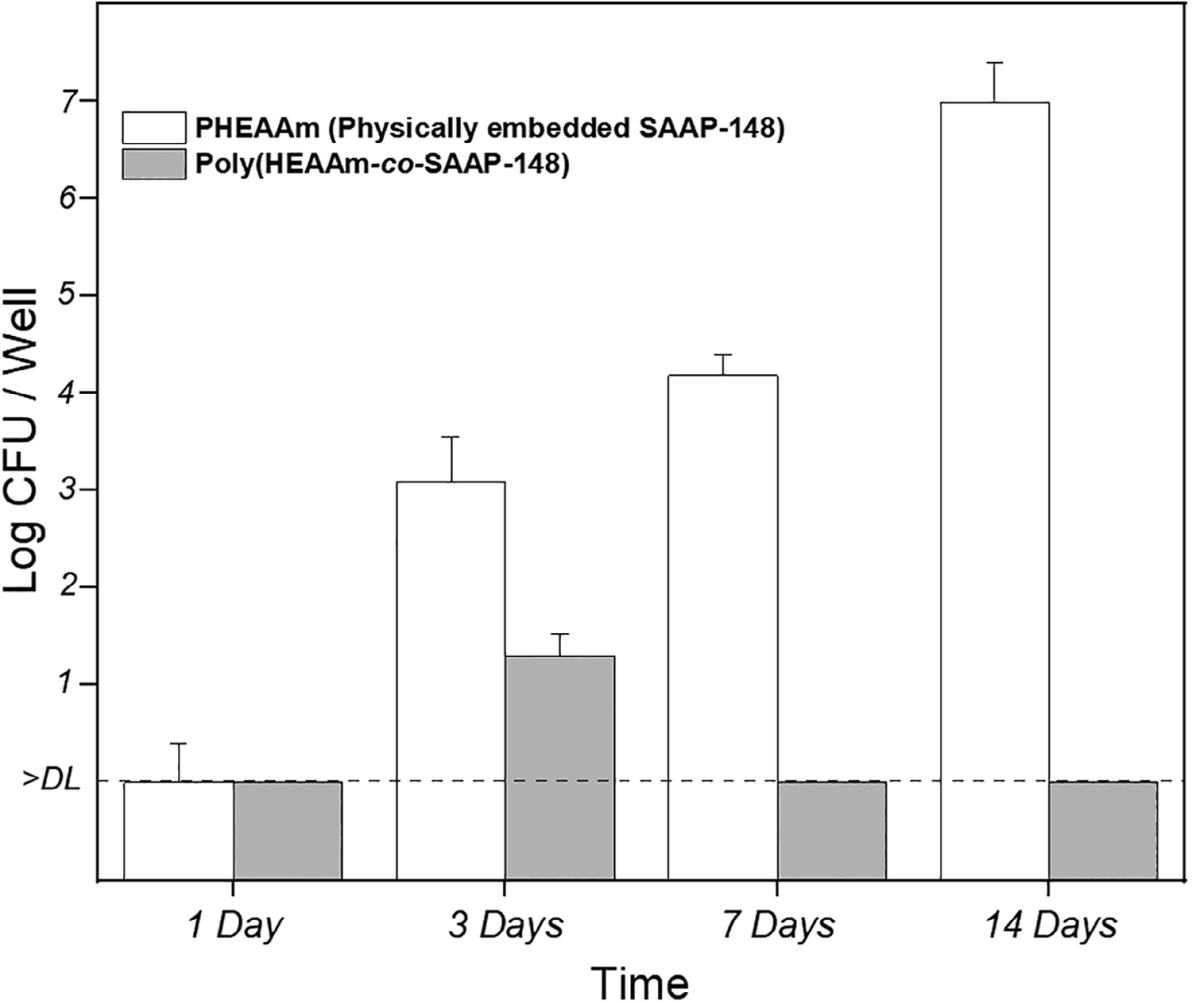
Comparison of microbicidal activity of PHEAAm with physically embed SAAP‐148 and of poly(HEAAM‐co‐SAAP‐148) (chemically bonded SAAP‐148) against *E. coli* DH5α. The inoculum was 10^5^ CFU per well. Values are mean numbers of CFU + SD and duplicate samples were used (*n* = 2). The lower limit of detection (DL) is 3.3 CFU per well.

A PBS washing test was conducted over a duration of two weeks to assess the long‐term stability of the covalently immobilized SAAP‐148 within a photocrosslinked polymer network structure. This structure, upon swelling with water, forms a hydrogel. The results were then compared with the physical embedding of SAAP‐148 in the hydrogel. The results indicated that the hydrogel with covalently bound SAAP‐148 retained its antibacterial activity against *E. coli* DH5α for up to 14 days. While the hydrogel containing physically embedded SAAP‐148 lost its antimicrobial activity after 3 days (**Figure** **4**).

Moreover, after 7 days no detectable antimicrobial activity of SAAP‐148 leached from the hydrogel matrix into the PBS (Figure S11, Supporting Information). This was evidenced by the fact that the number of CFU of *E. coli* DH5α observed in the PBS aliquots, which had been in contact with the hydrogel containing chemically immobilized SAAP‐148, was almost identical to the CFU count in the pure PBS control. Therefore, the chemically immobilized SAAP‐148 within the hydrogel remained stable and did not diffuse into the surrounding medium, suggesting effective retention of the antimicrobial agent within the hydrogel matrix.

These results highlight the advantage of covalent immobilizing SAAP‐148, as the SAAP‐148‐modified hydrogels retain their bactericidal activity for an extended period of time while no SAAP‐148 is leaching from the hydrogel matrix into the surrounding environment even after 14 days of PBS exposure.

### 3.6 Retention of Antimicrobial Properties of SAAP‐148‐Modified Hydrogels Upon Exposure to Human Blood Plasma

**Figure 5:**
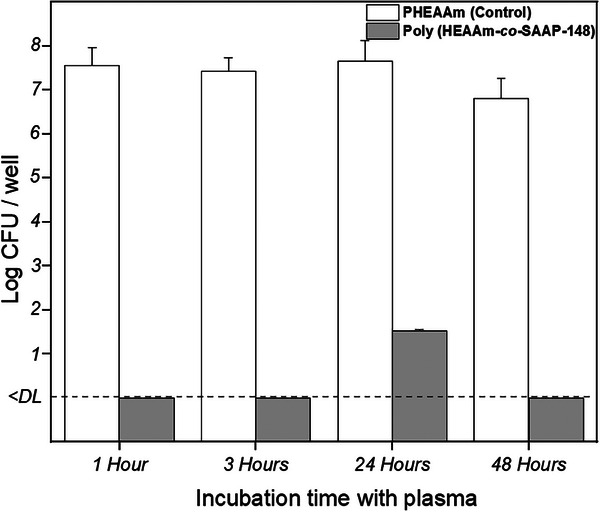
Analysis of the antimicrobial activity against *E. coli* DH5α for SAAP‐148‐modified hydrogels preincubated with 20% human blood plasma for 1–48 h, followed by an overnight incubation with bacteria (10^5^ CFU per well). Values are mean numbers of CFU + SD, duplicate samples were used (*n* = 2). The lower limit of detection (DL) is 3.3 CFU.

The retention of the antimicrobial properties of SAAP‐148‐modified hydrogels was determined in the presence of human blood plasma for *E. coli* as an example, since the stability of peptides in plasma or serum is considered an appropriate indicator for their stability in vivo.^[^ ^65^ ^]^ SAAP‐148‐free hydrogel (PHEAAm) was used as a control. The SAAP‐148‐modified hydrogel demonstrated its antimicrobial effect against *E.coli* DH5α after prior incubation of the hydrogel in human blood plasma for up to and including the last time point of 48 h, showing an almost complete elimination of the bacteria (a maximum of 33 CFU per well remaining after 24 h), To compare, with a hydrogel without SAAP‐148 as a control sample, an increase in numbers of CFU (6.3–6.8 log CFU per well) relative to the inoculum (10^5^ CFU per well) was observed. (**Figure** **5**).

Previous studies have shown that many AMPs are inactivated in the presence of plasma due to the inhibitory effects of plasma components.^[^ ^66^, ^67^, ^68^ ^]^ The loss of AMP activity in serum is likely due to two main factors: high ionic strength and the presence of proteins and enzymes in the serum. Cationic peptides having positively charged groups like ammonium functionalities after protonation of arginine and lysine are particularly susceptible. These charged groups act as cleavage sites for enzymes naturally present in blood, leading to AMP degradation.^[^ ^37^ ^]^

Covalent binding of AMPs to a carrier could improve their stability over 24 h when attached to an elastin‐like polypeptide matrix.^[^ ^69^ ^]^ Our results also show that attaching SAAP‐148 covalently to a hydrogel matrix retains its antimicrobial activity even after exposure to plasma. This observation complements previous studies on AMPs that are active while dissolved in blood plasma. SAAP‐148 was developed with activity in 50% human plasma as a criterium.^[^ ^45^ ^]^ In the present study, a fraction of the amino groups from the SAAP‐148 are involved in the covalent attachment to the hydrogel matrix via the active ester coupling strategy, which apparently does not hamper or may even contribute to the retention of the functionality even in the presence of blood plasma.

## 4 Conclusion

The present study describes the development of a novel antimicrobial hydrogel for potential application in wound dressing materials, which offer an alternative to conventional antibiotics for the treatment of skin wound infections. We have designed the antimicrobial copolymer poly(HEAAm‐*co*‐SAAP‐148) by covalent immobilization of the AMP SAAP‐148 at the active ester sites of the parent copolymer poly(HEAAm‐*co*‐BPAAm‐*co*‐PFPA‐*co*‐EH3A). The resulting polymer is not cytotoxic to human lung fibroblasts and the polymer solution showed antimicrobial activity against several strains of Gram‐positive and Gram‐negative bacterial species, i.e., of *E. coli, S. aureus, P. aeruginosa*, and *A. baumannii*. The polymer can be further transformed into a photocrosslinked hydrogel by irradiation with UV light and swelling with water. The covalently immobilized SAAP‐148 does not leach out to the surrounding medium and the hydrogel retains its antimicrobial functionality against *E. coli* even after incubation in 20% human blood plasma for at least 48 h. A previous study demonstrated the ability of SAAP‐148 in solution to kill *Clostridium difficile* under anaerobic conditions, to combat *S. aureus* and *A. baumannii* biofilms, as well as being highly effective against multidrug‐resistant (MDR) Gram‐positive and Gram‐negative ESKAPE pathogens.^[^ ^45^ ^]^ This broad‐spectrum bactericidal activity of SAAP‐148 in solution is expected to translate also to our contact‐killing strategy with SAAP‐148‐modified hydrogels and to reduce the development of antibiotic resistance by unnecessary antibiotic usage.

## Conflict of Interest

Yes. Patent application has been submitted on 31.10.2024 to the EPO with application number EP 24 210 082.4.

## Supporting information



Supporting Information

## Data Availability

The data that support the findings of this study are available from the corresponding author upon reasonable request.
