# Supplementary material for: Antimicrobial Peptide SAAP‐148‐Functionalized Hydrogels from Photocrosslinkable Polymers with Broad Antibacterial Activity
Source: Macromol Rapid Commun. 2024 Nov 12;45(24):2400785. doi: 10.1002/marc.202400785 (PMC11661662; doi:10.1002/marc.202400785)
Supplement: Supplementary file 1 — Supporting Information [file MARC-45-2400785-s001.pdf]

Macromolecular Rapid Communications

Supporting Information

for *Macromol. Rapid Commun.*, DOI 10.1002/marc.202400785

Antimicrobial Peptide SAAP-148-Functionalized Hydrogels from Photocrosslinkable Polymers with Broad Antibacterial Activity

*Muhammad Atif, Gizem Babuççu, Martijn Riool, Sebastian Zaat and Ulrich Jonas**

Antimicrobial Peptide SAAP-148-Functionalized Hydrogels from Photocrosslinkable Polymers with Broad Antibacterial Activity

Muhammad Atif [1], Gizem Babuçcu [2], Martijn Riool [2,3], Sebastian Zaat [2], and Ulrich Jonas* [1]

[1] Macromolecular Chemistry, Department of Chemistry and Biology, University of Siegen, Adolf-Reichwein-Strasse 2, 57076, Siegen, Germany.

[2] Department of Medical Microbiology and Infection Prevention, Amsterdam UMC, Amsterdam institute for Immunology and Infectious Diseases, University of Amsterdam, Meibergdreef 9, 1105 AZ, Amsterdam, The Netherlands.

[3] Laboratory of Experimental Trauma Surgery, Department of Trauma Surgery, University Hospital Regensburg, Am Biopark 9, 93053, Regensburg, Germany.

* Corresponding author: Ulrich Jonas (jonas@chemie.uni-siegen.de)

1. Synthesis of ECOSURF (EH3A) surfactant monomer

ECOSURF acrylate (EH3A) was synthesized following the procedure given in the literature.(1)

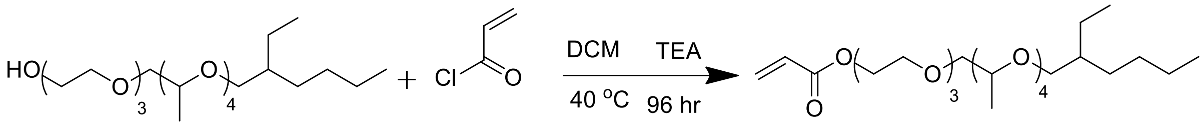

Scheme S1. Synthesis of EH3 acrylate

EH3A (9.61 mL, 18.76 mmol) was dissolved in dichloromethane (20 mL) in a two necked flask with a magnetic stirring bar inside. Triethylamine (TEA) in dichloromethane (20 mL) was added and the reaction mixture was stirred for one hour in an ice bath. Afterwards a solution of prop-2-enoyl chloride in dichloromethane (40 mL) was added under argon dropwise with strong stirring. The reaction mixture was purged with argon, sealed, and stirred under the reflux condenser for 96 hours. To purify the reaction, the mixture was first neutralized with aqueous sodium hydroxide (0.1 M, 3 mL), and the solvent was removed. The residue was taken in an aqueous sodium hydroxide (1 M, 100 mL) and the aqueous phase was washed with dichloromethane (2x, 50 mL). In the next step the organic phases were combined, dried with magnesium sulphate and the solvent was evaporated. The residue was dissolved in dichloromethane (100 mL) and washed with aqueous hydrochloride

solution (2x, 1 M, 50 mL). The aqueous phase was extracted with dichloromethane (50 mL) and the organic phases were combined, dried with magnesium sulphate and the solvent was evaporated. The slightly yellow viscous liquid having the yield of 87% was dried in a vacuum overnight.

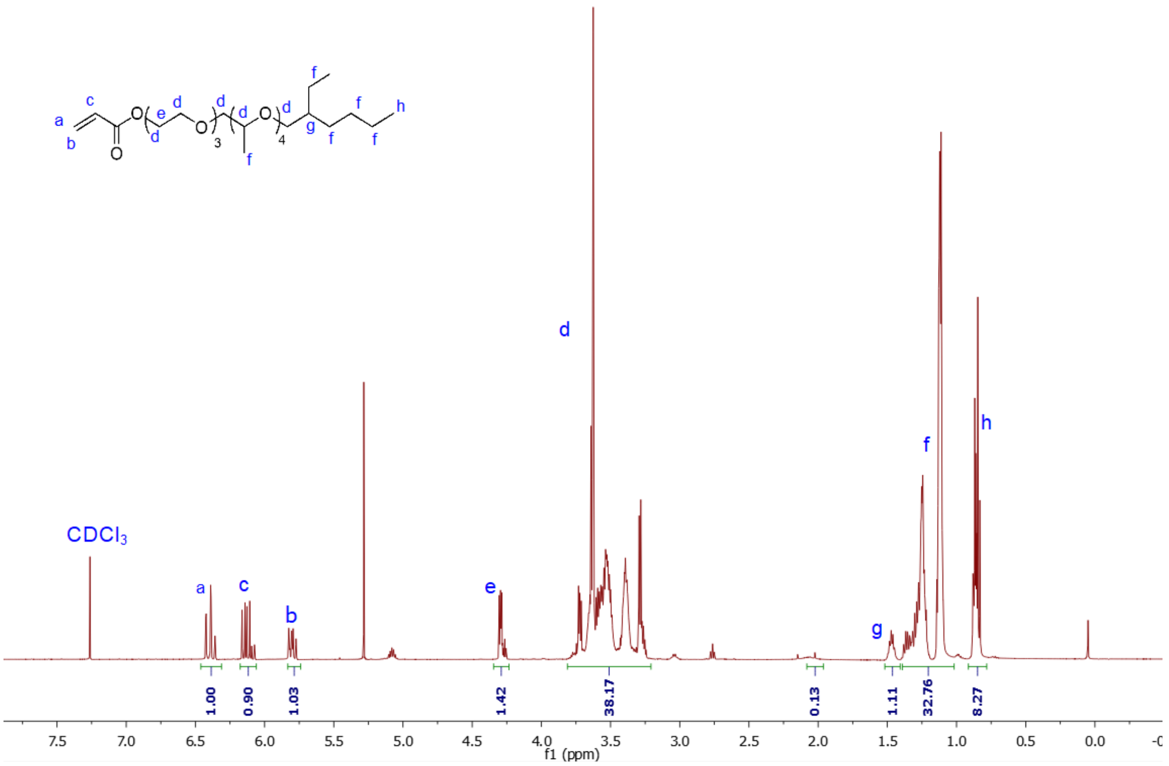

Figure S1. ^{1}H NMR of ECOSURF acrylate (EH3A) monomer (in $CDCl_3$).

2. Synthesis of 4-benzophenone acrylamide (BPAAm) photo crosslinker

BPAAm was synthesized according to the procedure given in the literature.(2)

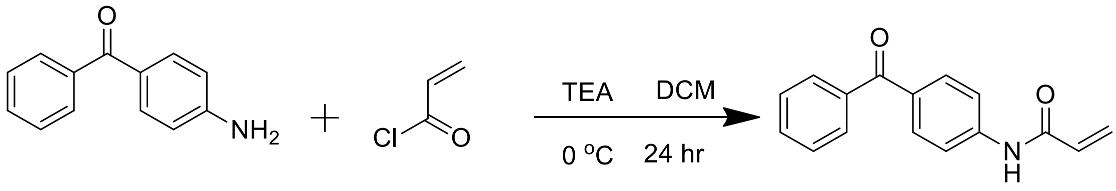

Scheme S2. Synthesis of BPAAm.

4-amino benzophenone (1.97 g, 10.0 mmol) was dissolved in dichloromethane (80 mL) containing Potassium carbonate (1.66 g, 12.0 mmol) in a two necked flash with a

magnetic stirring bar inside. Prop-2-enoyl chloride (0.98 mL, 12.0 mmol) was dissolved in dichloromethane (50 mL) and added to a dropping funnel. The solution was added dropwise to the reaction mixtures, which was cooled with ice under vigorous stirring. After 48 h, the product was cleaned with column chromatography (silica gel, CH_2Cl_2: EtOAc = 8:1). The product was separated by the side product by using a TLC (silica gel, CH_2Cl_2: EtOAc = 8:1). Afterwards the slightly brownish solid was dried with vacuum overnight having a yield of 69%.

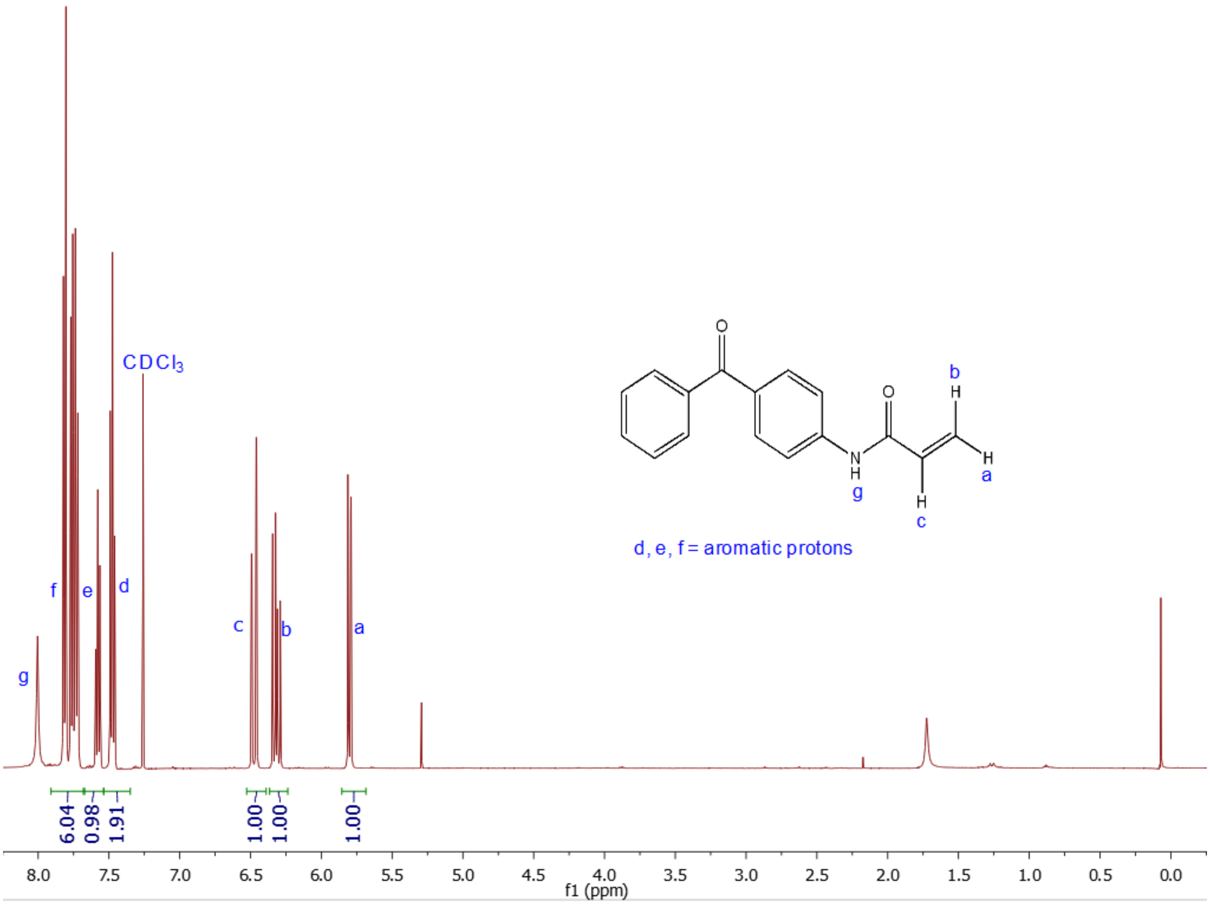

Figure S2. ^{1}H NMR of 4-benzophenone acrylamide (in $CDCl_3$)

3. Synthesis of pentafluorophenyl acrylate as an active ester monomer

Pentafluorophenyl acrylate (PFPA) was synthesized according to the procedure given in the literature.(3)

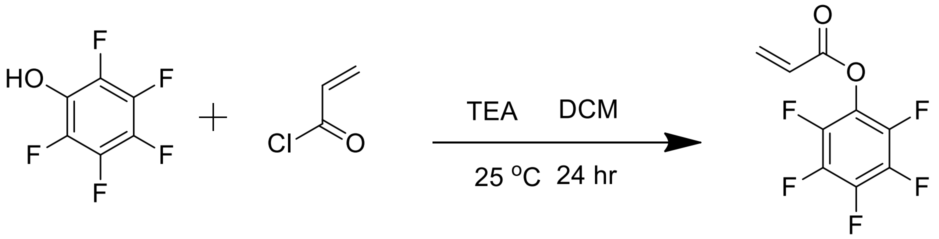

Scheme S3. Synthesis of PFPA

Pentafluorophenyl (10.0 g, 54.3 mmol) was dissolved in dichloromethane (~ 90 mL) and cooled to 0 °C. Triethylamine (8 mL, 5.8 g, 1 mol eq.) was added and a mixture of acryloyl chloride (5 mL, 5.6 g) in dichloromethane (15 mL) was slowly added dropwise. Another 100 mL of DCM was added, and the mixture was stirred overnight at room temperature (~ 20 °C). The mixture was extracted with brine (3x, ~ 150 mL), dried over sodium sulphate, the solvent was removed under reduced pressure and the product was finally dried under vacuum (< 1 mbar) and obtained colourless liquid having a yield of 85%.

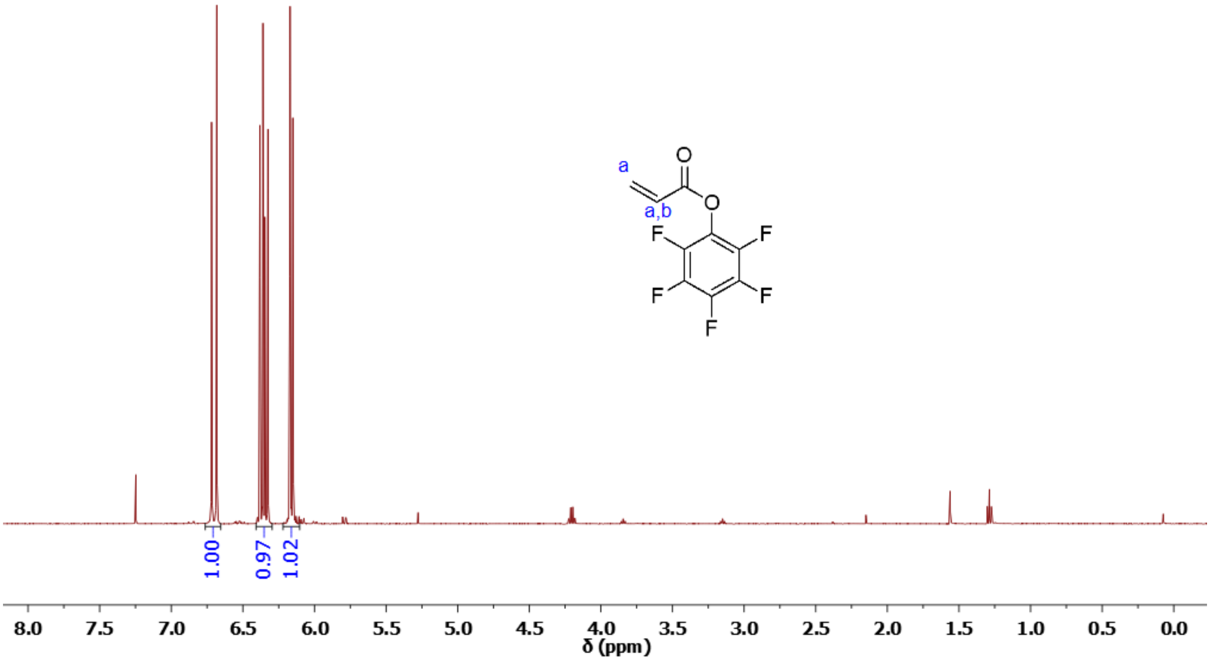

Figure S3. ^{1}H NMR of pentafluorophenyl acrylate (in $CDCl_3$).

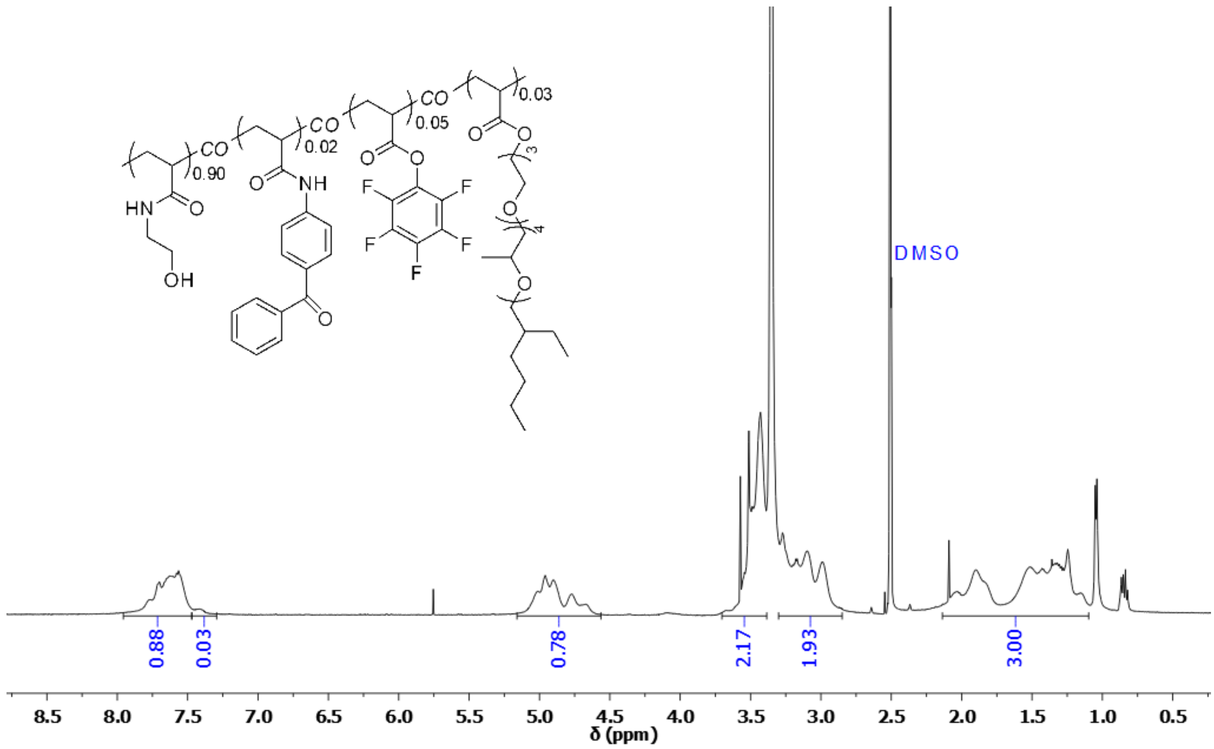

Figure S4. ^{1}H NMR of poly(HEAAm-co-BPAAm-co-PFPA-co-EH3A).

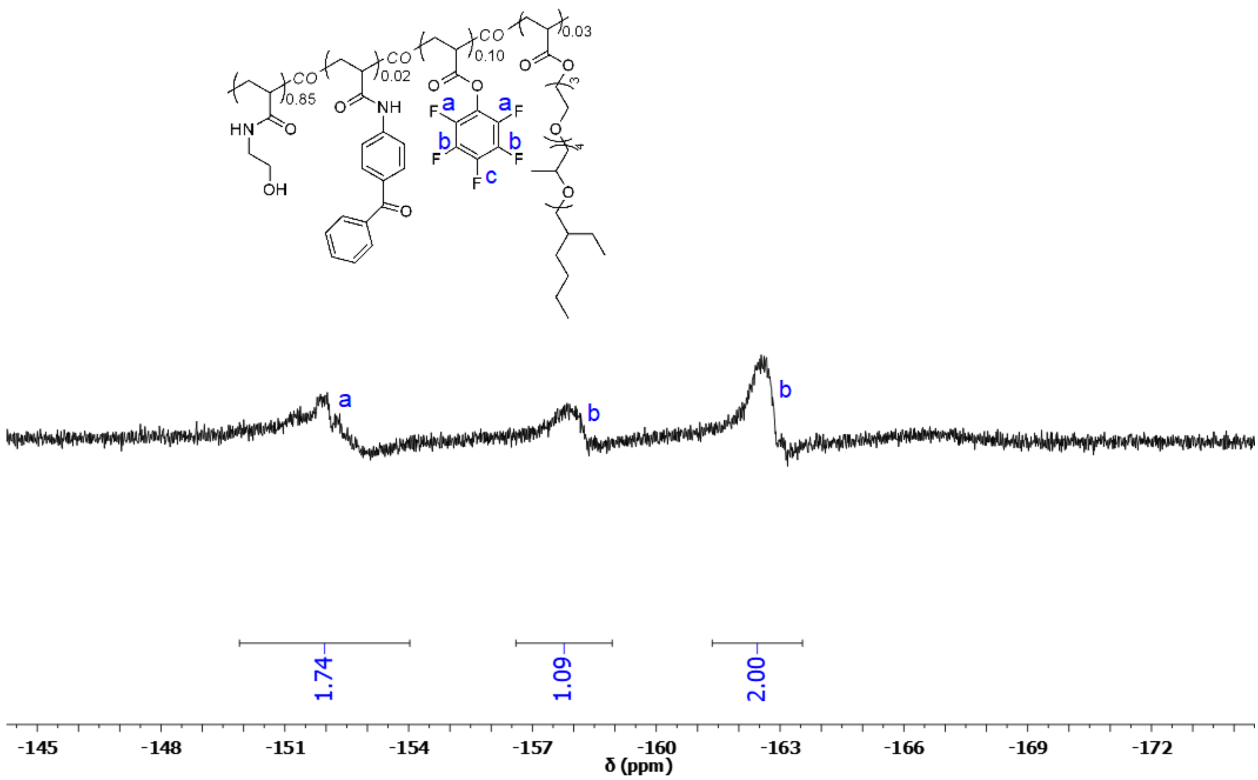

Figure S5. ^{19}F NMR of poly(HEAAm-co-BPAAm-co-PFPA-co-EH3A).

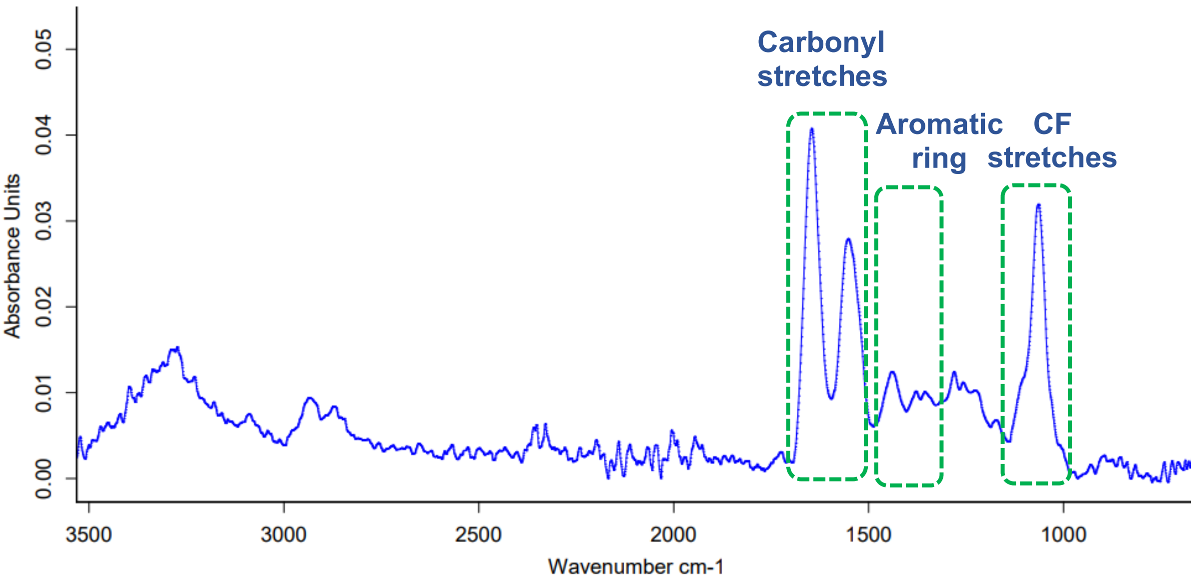

Figure S6. FTIR of poly(HEAAm-co-BPAAm-co-PFPA-co-EH3A)

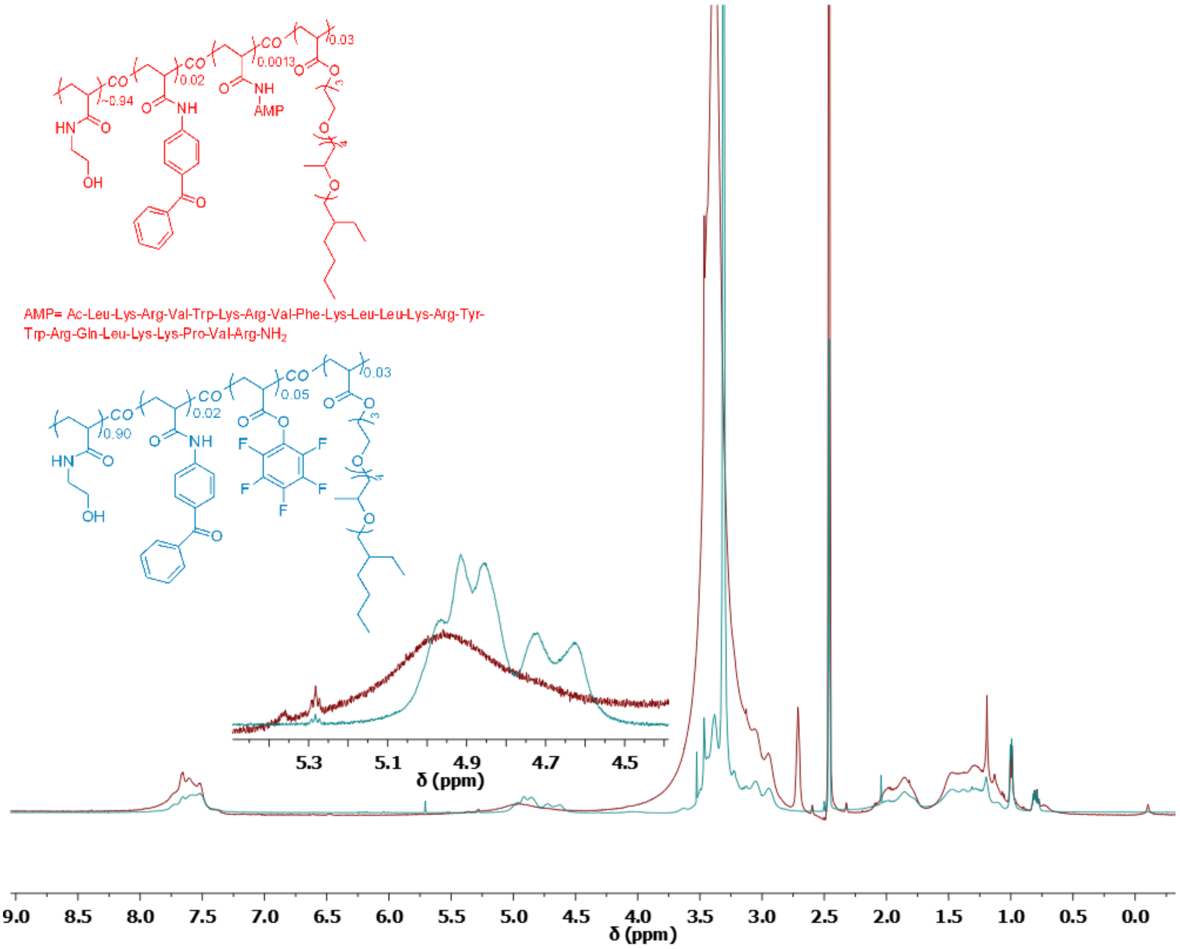

Figure S7. Comparison of ^{1}H NMR of poly(HEAAm-co-BPAAm-co-PFPA-co-EH3A) (blue spectrum) and poly(HEAAm-co-BPAAm-co-SAAP-148-co-EH3A) (red spectrum).

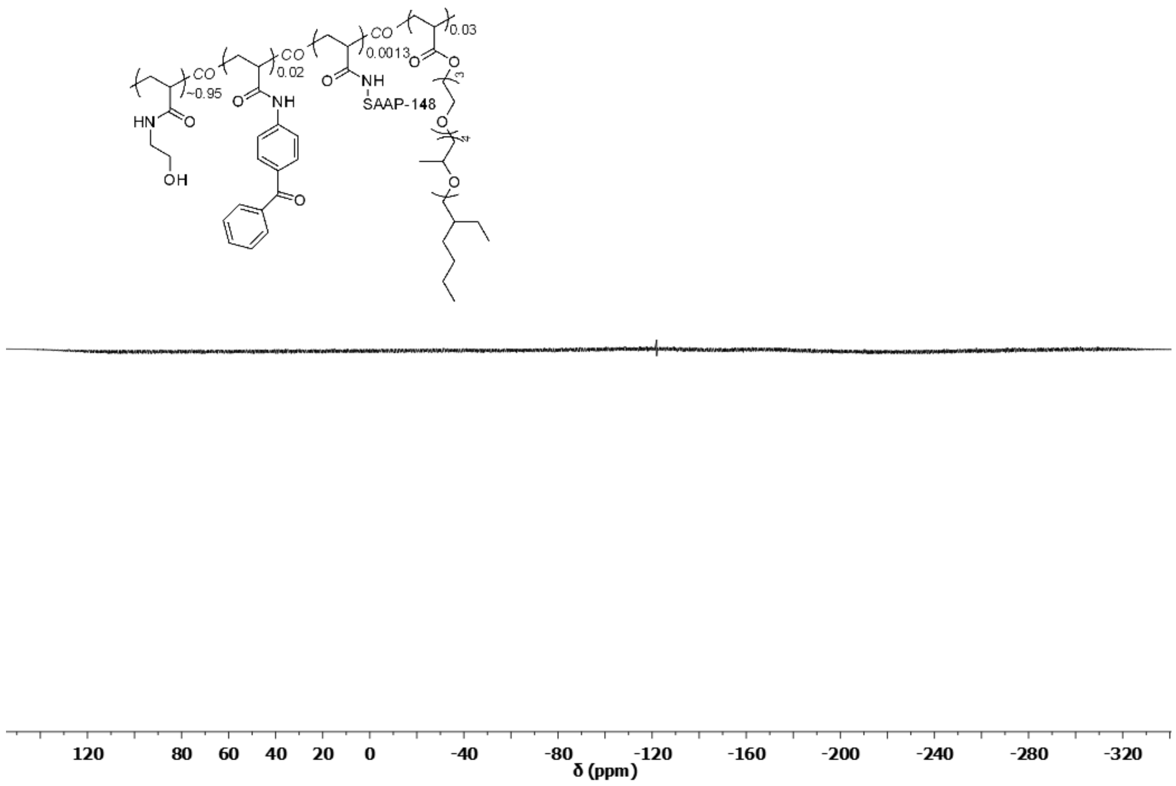

Figure S8. ^{19}F NMR of poly(PHEAAm-co-BPAAm-co-SAAP-148-co-EH3A).

(a)

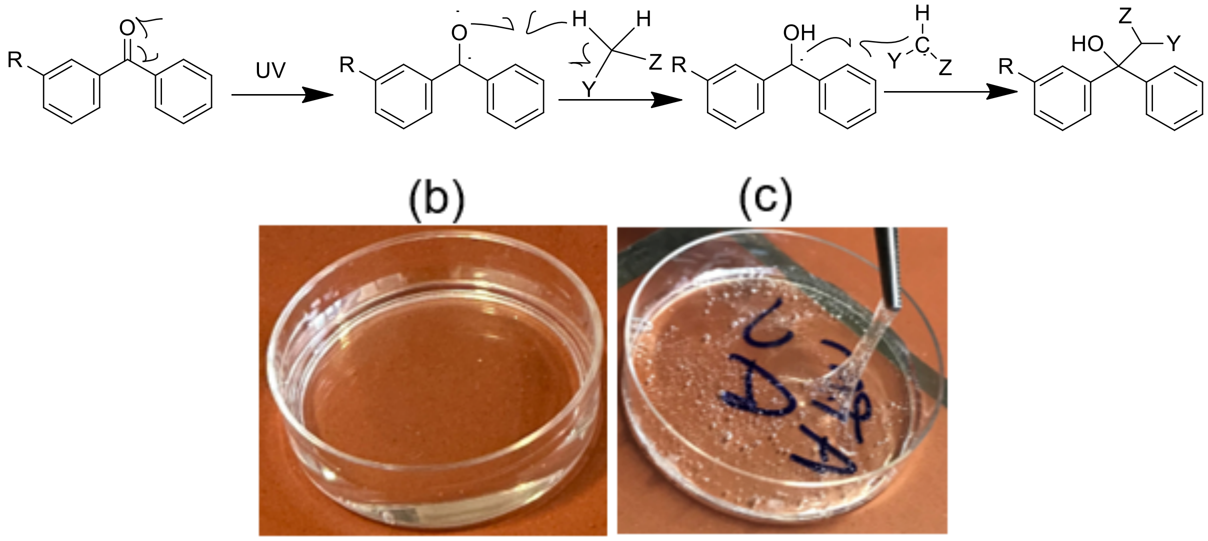

Figure S9. (a) Mechanism of photocrosslinking reaction of benzophenone derivatives under UV light. (b) Polymer solution before photocrosslinking (c) hydrogel formation after photocrosslinking.

.

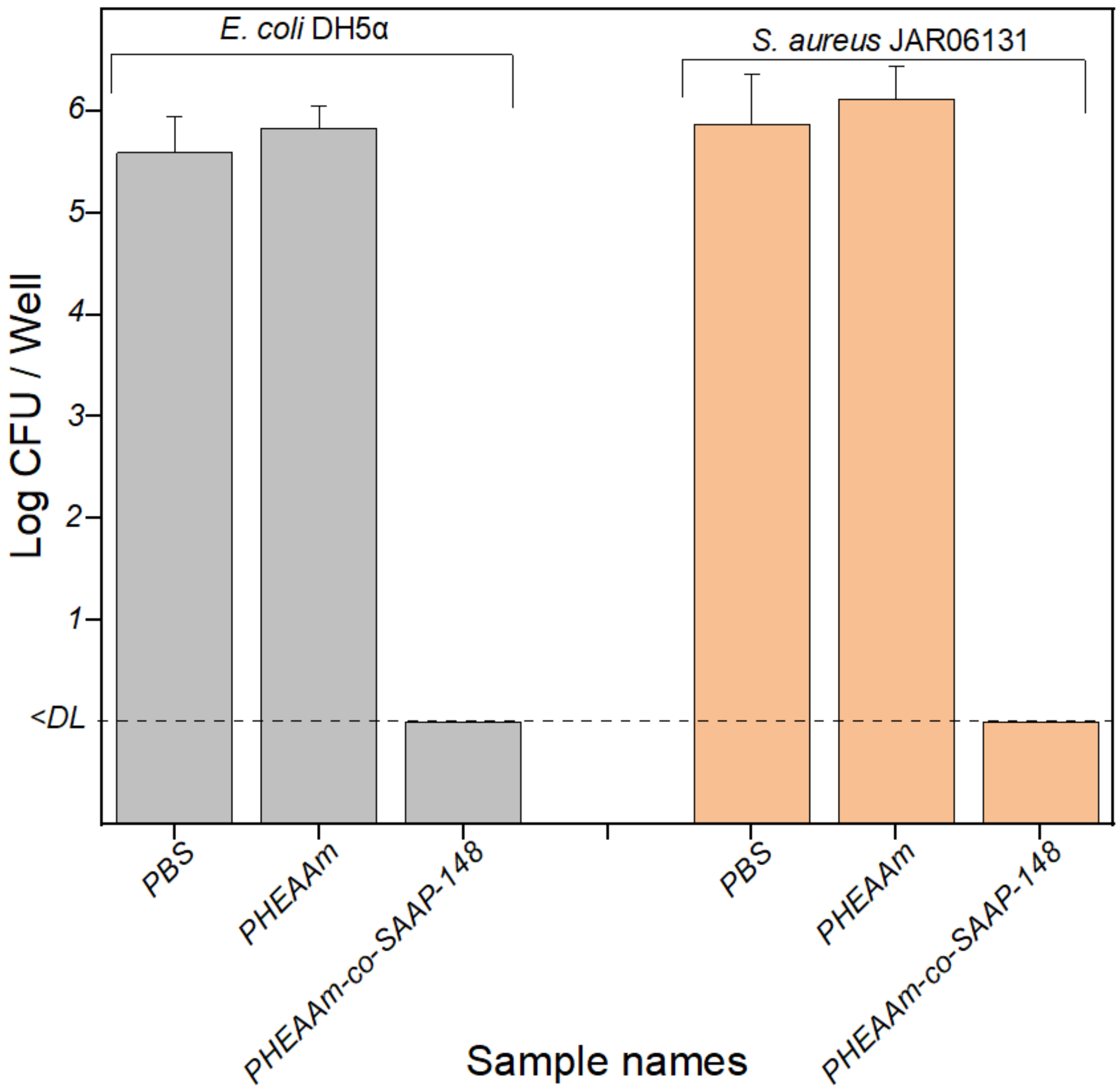

Figure S10. Analysis of the antimicrobial properties of different polymers in solution against E. coli DH5α and S. aureus JAR06131 strains after 2 hours incubation time. Values are mean numbers of CFU + SD, duplicate samples were used, and two independent experiments were performed (n=2). The lower limit of detection (DL) is 6.6 CFU.

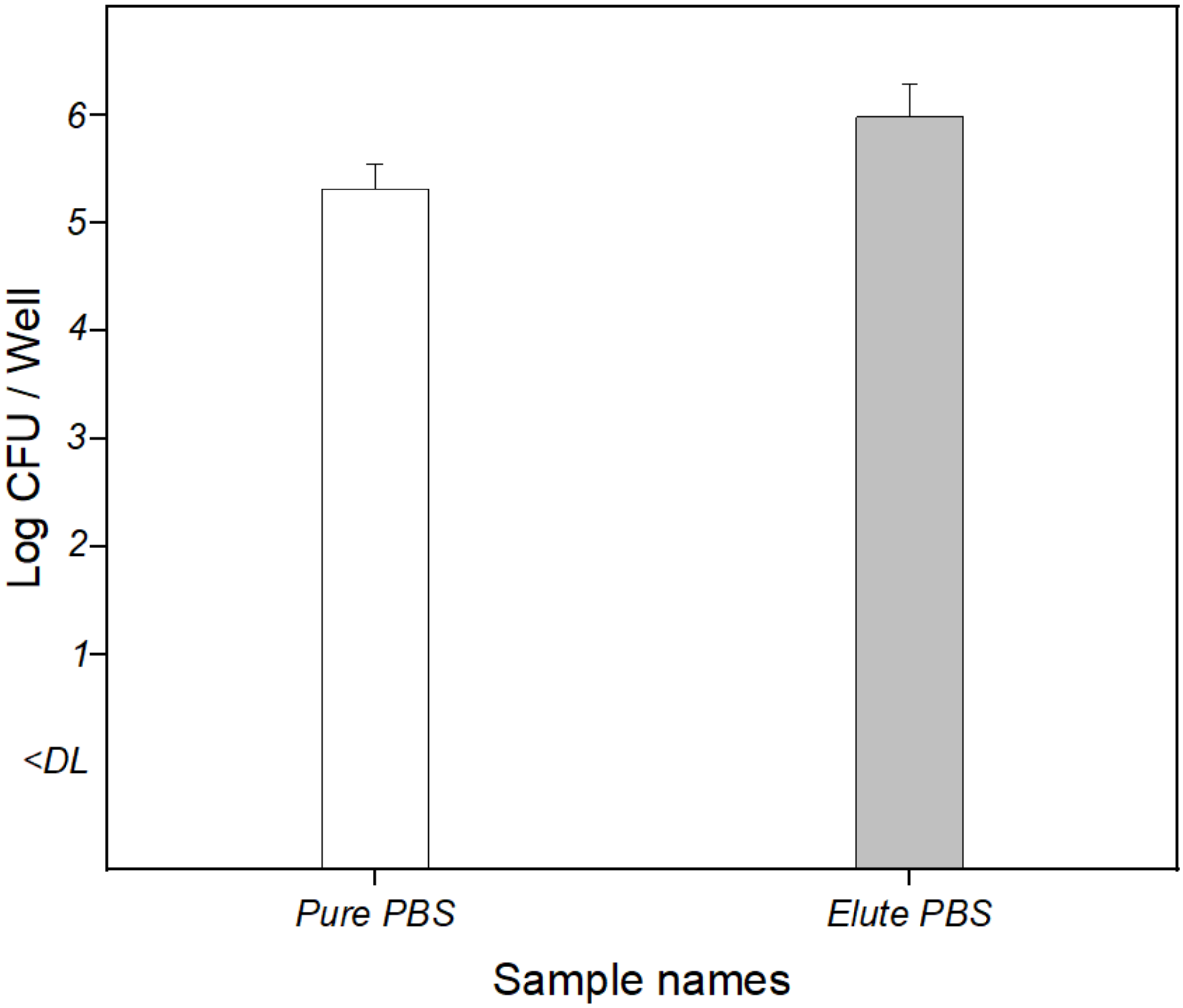

Figure S11. Analysis of leaching out of SAAP-148 from chemically bonded SAAP-148 in the hydrogel against E. coli DH5α. Pure PBS was taken as control, while the washed-out PBS was collected after rinsing the SAAP-148 immobilized hydrogel. Values are mean numbers of CFU + SD and duplicate samples were used (n=2). The lower limit of detection (DL) is 6.6 CFU.

References

1. Freese S, Diraoui S, Mateescu A, Frank P, Theodorakopoulos C, Jonas U. Polyolefin-supported hydrogels for selective cleaning treatments of paintings. Gels. 2019;6(1):1.
2. Peng B, Tong Z, Tong WY, Pasic PJ, Oddo A, Dai Y, et al. In situ surface modification of microfluidic blood–brain-barriers for improved screening of small molecules and nanoparticles. ACS Applied Materials & Interfaces. 2020;12(51):56753-66.
3. Theato P. Synthesis of well-defined polymeric activated esters. Journal of Polymer Science Part A: Polymer Chemistry. 2008;46(20):6677-87.
